# Validation of a Virtual Assistant for Improving Medication Adherence in Patients with Comorbid Type 2 Diabetes Mellitus and Depressive Disorder

**DOI:** 10.3390/ijerph182212056

**Published:** 2021-11-17

**Authors:** Surya Roca, María Luisa Lozano, José García, Álvaro Alesanco

**Affiliations:** 1 Aragón Institute of Engineering Research (I3A), University of Zaragoza, 50018 Zaragoza, Spain; jogarmo@unizar.es (J.G.); alesanco@unizar.es (Á.A.); 2 Centro de Salud Las Fuentes Norte, 50002 Zaragoza, Spain; mllozano@salud.aragon.es

**Keywords:** chat-based interaction, eHealth, mHealth, chronic disease, diabetes, depressive disorder, messaging platforms, medical virtual assistant, health information technology, chronic patient support

## Abstract

Virtual assistants are programs that interact with users through text or voice messages simulating a human-based conversation. The development of healthcare virtual assistants that use messaging platforms is rapidly increasing. Still, there is a lack of validation of these assistants. In particular, this work aimed to validate the effectiveness of a healthcare virtual assistant, integrated within messaging platforms, with the aim of improving medication adherence in patients with comorbid type 2 diabetes mellitus and depressive disorder. For this purpose, a nine-month pilot study was designed and subsequently conducted. The virtual assistant reminds patients about their medication and provides healthcare professionals with the ability to monitor their patients. We analyzed the medication possession ratio (MPR), measured the level of glycosylated hemoglobin (HbA1c), and obtained the patient health questionnaire (PHQ-9) score in the patients before and after the study. We also conducted interviews with all participants. A total of thirteen patients and five nurses used and evaluated the proposed virtual assistant using the messaging platform Signal. Results showed that on average, the medication adherence improved. In the final interview, 69% of the patients agreed with the idea of continuing to use the virtual assistant after the study.

## 1. Introduction

Diabetes is a major health issue, affecting 463 million people worldwide, and is expected to increase to 700 million by 2045 [1]. Patients with type 2 diabetes require a complex self-management of different aspects of their lives (e.g., exercise, diet, medication, or blood glucose control) [2,3]. The relationship between diabetes and depression has been widely studied [4,5] and it has been demonstrated that there exists a highly possible bidirectional relationship between them [6]. Depression is a common disease that affects more than 268 million people worldwide [7]. Patients with depression have mood fluctuations that affect their daily lives. These patients have problems in handling challenges at work, with family, or at school. This serious health disease, characterized by persistent sadness, is more frequent in patients with chronic diseases in general, and diabetes in particular [8], and is associated with poor medication adherence in patients with comorbidities [9].

The use of text messages to improve medication adherence is not new. Yasmin et al. [10] found that using mobile short message systems and/or voice calls improves patients’ adherence. The habits of people in relation to technology have changed dramatically during the last decade. Messaging platforms are now essential for people’s interaction and new mHealth platforms aimed at improving patients’ lives must be aware and take advantage of this fact. The combination of messaging platforms and virtual assistants provides a new scenario in healthcare to align eHealth tools and services to patients and physicians. Moreover, messaging platforms have become a widespread communication channel and the use of cloud technologies with their high computational and storage capacity allows them to deploy virtual assistants with more advanced techniques, such as artificial intelligence and other computationally heavy algorithms.

Virtual assistants are widely used in mHealth scenarios to offer help and advice to patients. Nevertheless, most current virtual assistants are app-based, such as eMMA [11], which manages patient medication through a chatbot focused on increasing medication adherence. However, the development of virtual assistants, which use messaging platforms is rapidly increasing. MamaBot [12] uses Telegram to provide support to mothers, pregnant women, and families with young children. Custom-RXBot [13] is a chatbot developed to help with personalized prescriptions for dermatology through Slack. Roborto [14] uses Telegram to inform healthcare providers about patients’ conditions and improvements, and tries to improve patients’ medication adherence. Roca et al. [15] proposed a virtual assistant prototype, which uses Signal, to provide medication management. However, the usage of our proposed virtual assistant prototype has yet to be evaluated to determine whether it improves patients’ adherence and reduces medical appointments. This study intends to do precisely that.

Earlier studies have tested artificial conversational agents in relation to different diseases, such as cancer, chronic pain, or coronary heart disease [16,17,18]. Some of these studies examined the use of virtual assistants to improve adherence in patients with breast cancer, coronary artery disease, and other chronic diseases [19,20,21]. Several studies have suggested that mobile phone text messaging interventions could have considerable potential to improve medication adherence in patients with chronic disease [22]. Studies focused on patients with type 2 diabetes mellitus using mobile applications showed that there was a moderate effect on glycemic control, with an overall difference in the mean HbA1c of −0.40% [23]. Most of these apps are focused on measuring blood-glucose using external devices in combination with a smartphone or a web-based interface. Previous depression studies have focused on providing patients with a therapeutic resource through conversational agents [24,25]. Sarda et al. [26] studied the relationship between smartphone-sensing parameters and symptoms of depression in patients with depression and diabetes. Bogner et al. [27] improved the medication adherence of patients with type 2 diabetes and depression using the medication event monitoring system (MEMS). To the best of the authors’ knowledge, no studies have been carried out related to improving adherence using virtual assistants in combination with messaging platforms for comorbid patients with type 2 diabetes mellitus and depressive disorder.

To fill this knowledge gap, this study focuses on a virtual assistant for patients with type 2 diabetes mellitus and depressive disorder, with the aim of improving medication adherence through medication reminders. The study examined medical variables (the level of glycosylated hemoglobin (HbA1c), the patient health questionnaire (PHQ-9), the medication adherence, and the number of medical appointments), as well as the acceptance and real use of the virtual assistant to validate our approach.

## 2. Materials and Methods

### 2.1. Virtual Assistant

**Figure 1:**
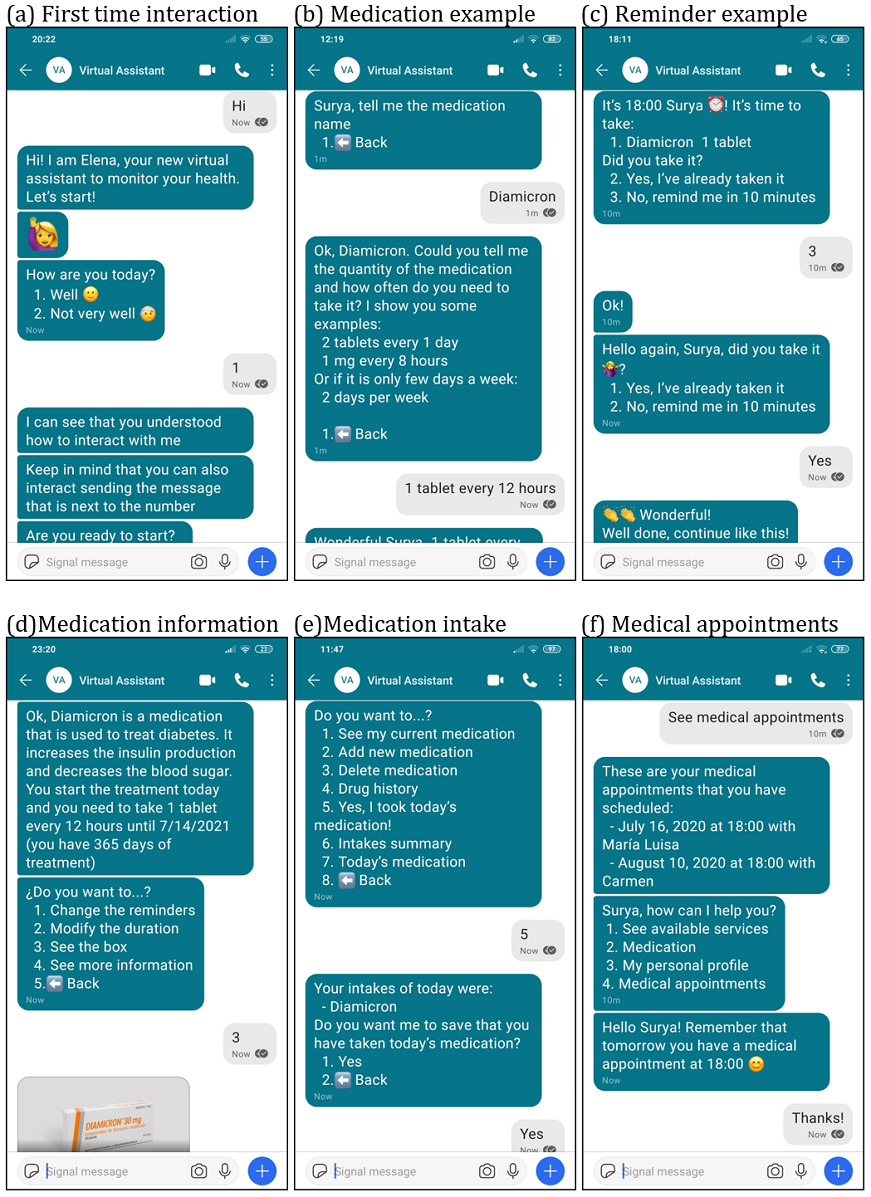
Screenshot of real interactions with the virtual assistant.

The technical aspects and the internal architecture of the virtual assistant used in this study are based in the work described by Roca et al. [28]. The virtual assistant is developed using a microservice architecture, storing all the medical data in accordance with the Health Level 7 Fast Healthcare Interoperability Resources (FHIR) clinical standard. All communications and data storage are secure and private, following the General Data Protection Regulation (GDPR). User conversation (patient to virtual assistant and vice versa) is defined using Artificial Intelligence Markup Language (AIML). Examples of the user to virtual assistant conversations along with the user interface for interaction are illustrated in Figure 1 and are self-explanatory by simply reading the users’ interactions. The way the patient interacts with the virtual assistant is very simple: the virtual assistant offers some choices to the patient and the patient indicates his/her selection either by writing the number that identifies the choice (numeric-based message) or by typing the text of the choice (text-based message). As described by Roca et al. [15], the most secure messaging platform to use in this study is Signal [29]. The choice was driven by its security characteristics: end-to-end encryption and central servers’ privacy.

The functionalities developed for this medication adherence study were discussed and designed in collaboration with the nurses from the primary healthcare center Las Fuentes Norte in Zaragoza, Spain, who were in charge of the recruited patients. These functionalities are described in Table 1. The first meetings with the nurses were about what they think will be useful to have in a virtual assistant based on their professional experience monitoring patients. After showing them the prototype, a few more meetings were held in order to improve the chatbot conversation and to obtain the final version of the virtual assistant. The two main functionalities included are “medication option”, where patients can add medication and medication reminders, and “appointment option”, where patients can add an appointment with their healthcare professionals and configure appointment notifications. Medication reminders are sent a maximum of three times per programmed intake. If the patient does not respond to the reminder, another reminder is scheduled with a delay of 10 min. If the patient has not answered after three reminders, the virtual assistant stops reminding the patient and marks the intake as unanswered. Nevertheless, the virtual assistant includes a way for the patient to indicate that despite not answering the reminders, the medication was taken. This is a very useful safeguard because it could be a common situation for the patient not to hear the reminders (thus not answering on time), but taking the medication nevertheless. Thus, as we can observe in Figure 1c,e, the patient can inform the virtual assistant that the medication was taken by two options: either by answering the reminders or by telling the virtual assistant using the medication options menu. In addition, the virtual assistant shows the weather forecast every morning, gives a summary of the adherence every week, and sends links to tutorials on YouTube that explain how to use the virtual assistant. It was included an initial tutorial where the basic usage of the virtual assistant was explained.

### 2.2. Initial Setup

We designed and implemented a nine-month pilot study with patients with comorbid type 2 diabetes mellitus and depressive disorder to test a virtual assistant developed to improve medication adherence. Participants received detailed information about the study, the procedure, the virtual assistant and their privacy and anonymity. A document containing all the information and contact details of the research team was given to the participants. Once they acknowledged that they had understood the information, a written and signed informed consent was obtained from all the participants of the study (patients and healthcare professionals). When the participants interacted with the virtual assistant for the very first time, they needed to confirm that they had signed the written consent to participate in this study.

The research team explained to the nurses how to use and configure the virtual assistant. The nurses then explained and configured the virtual assistant for their patients during their medical appointments. The initial configuration consisted of assistance in downloading the Signal app, explaining the first interaction with the virtual assistant, the registration of the patient in the platform, and the configuration of the medication and the reminder functions.

### 2.3. Participants

Eligible participants were patients with type 2 diabetes and depressive disorder who were 18 years old or older. The reference code of the diseases listed in the *International Classification of Primary Care* (2nd Edition) are T90 (type 2 diabetes) and P76 (depressive disorder). The participants in the experiment were recruited from the primary healthcare center Las Fuentes Norte in Zaragoza, Spain. Potential patients were recruited by the nurses working in the primary healthcare center. They were asked in their regular visits to the healthcare center to confirm their availability and willingness to participate. The experimental research design was a pre/post design, i.e., a comparison of outcomes in the same group of patients before and after the planned intervention of the virtual assistant.

The inclusion criteria of the patients were the following:

- Patients must have regular appointments with the nurses.
- Patients need to take medication every day.
- Patients have poor medication adherence. Poor adherence is measured with the level of the medication possession ratio (MPR). MPR is the division between the number of drug units prescribed for a specific period divided by the number of days [30]. The MPR value is capped at 100%. A presence/absence of medication adherence is calculated with a binary variable. When the MPR value is below 80%, the medication adherence is considered as absence [31].
- Patients can read and understand Spanish.
- Patients have the ability to write a message in a messaging platform.
- Patients need to have a smartphone with Android or iOS, and they need to have access to the internet in their smartphones.
- Excluded from the study were subjects with cognitive, visual, or physical impairments that would interfere with the use of the virtual assistant.

The inclusion criteria of the healthcare professionals were the following:

- Healthcare professionals have a clinical interview experience.
- Healthcare professionals receive regular visits from patients with type 2 diabetes and depressive disorder.
- Healthcare professionals can read and understand Spanish.
- Healthcare professionals have the ability to write a message in a messaging platform.
- Healthcare professionals need to have a smartphone with Android or iOS, and they need to have access to the Internet in their smartphones.
- Excluded from the study were subjects with cognitive, visual, or physical impairments that would interfere with the use of the virtual assistant.

### 2.4. Medical Outcomes Measures

The medical effectiveness of the virtual assistant was measured by the level of glycosylated hemoglobin (HbA1c) in patients, which gives the mean level of blood glucose for the previous three months. The information provided by HbA1c allows the progression of the patients in the management of their diabetes to be evaluated. The higher the HbA1c values, the higher the risk of having complications related to diabetes. HbA1c measurements were obtained from a blood test done in a laboratory. The depression was measured using the patient health questionnaire (PHQ-9) [32], which monitors the severity of depression and the response to a medical treatment. Higher values in the PHQ-9 score indicate more severe depressive symptoms. The medication adherence was evaluated using the MPR value, with 80% as the threshold. The impact on healthcare resources was evaluated with the number of medical appointments per month for each patient. All the medical outcomes measurements were obtained at baseline and after 9 months.

### 2.5. Use Outcomes Measures

The patients’ use and acceptance of the virtual assistant were measured as follows: (a) the use of the virtual assistant was measured by the number of interactions every day. The type of interaction was studied in order to ascertain which kind of interaction the patients prefer (numeric or text-based interaction). (b) The use of tools was measured with the number of functionalities used every day. (c) The use of reminders was measured by the number of reminders answered. (d) The acceptance was measured by the number of patients that did not uninstall Signal. (e) The usefulness or otherwise of the assistant was measured by the number of times the patient was not understood by the virtual assistant. All use outcome measurements were taken 9 months after the start of the study.

### 2.6. Participant Interviews

Two sets of interviews were conducted during the study. The first interviews were conducted to obtain the patients’ opinions after three months of interaction. The second interviews were held to obtain the results and the final opinion of the participants.

In the first set of interviews, patients were asked for their opinions three months after the outset of the study. The interviews were designed to obtain the general impressions of the patients as well as to identify any issues and the suggestions for the improvement of the virtual assistant. An important objective was to gather ideas for the content of the YouTube tutorials to help patients in the use of the virtual assistant. These guided interviews took place in the primary healthcare center during the appointments of the patients with the nurses involved in the study.

The second set of interviews were conducted at the end of the study in order to obtain information about the HbA1c values, the PHQ-9 scores, the number of medical appointments and the opinion of the participants. During these post-study interviews, patients and healthcare professionals were asked their overall opinions about the study. The answers with multiple options for the patients were weighted with the following scale: always (5), almost always (4), sometimes (3), rarely (2), and never (1). The answers with multiple options for the healthcare professionals were weighted with the following scale: all the patients (5), almost all the patients (4), some patients (3), almost no patient (2) no patient (1). The patient questionnaire was focused on obtaining the differences in medication intake before and after the use of the virtual assistant, and the usefulness of the virtual assistant in their lives. This questionnaire was completed via telephone calls made by the nurses involved in the study. The healthcare professionals’ questionnaire included a question about their perception of the medication intake improvement of their patients. Interviews with healthcare professionals were self-administered.

The intermediate and post-study interview questions are provided in Multimedia Appendices Appendix A and Appendix B.

### 2.7. Ethical Aspects

The study protocol was approved and registered by the Comité de Ética de la Investigación de la Comunidad Autónoma de Aragón (CEICA) [33] on 13 March 2019 (minutes nº 05/2019). The CEICA committee acts in accordance with the Declaration of Helsinki (last modified in 2013) and with the Good Clinical Practice (GCP) standard. The study complied with both national data protection law LO 03/2018 [34] and European GDPR [35], providing the required measures of privacy and users’ rights.

### 2.8. Statistical Analysis

In order to describe continuous variables, mean and standard deviation (SD) were calculated, and to describe categorical variables, frequency and percentage were used. To compare the outcomes before and after the usage of the virtual assistant, the McNemar’s test was estimated for dichotomous variables (MPR score) and the Wilcoxon signed-rank test for continuous variables (HbA1c values, PHQ-9 score, medical appointments per month and post-study interview). Variables measured before and after were considered significantly different when the *p*-value was less than 0.05. All statistical analyses were conducted using R software, version 4.0.1 [36].

## 3. Results

### 3.1. Participants

**Figure 2:**
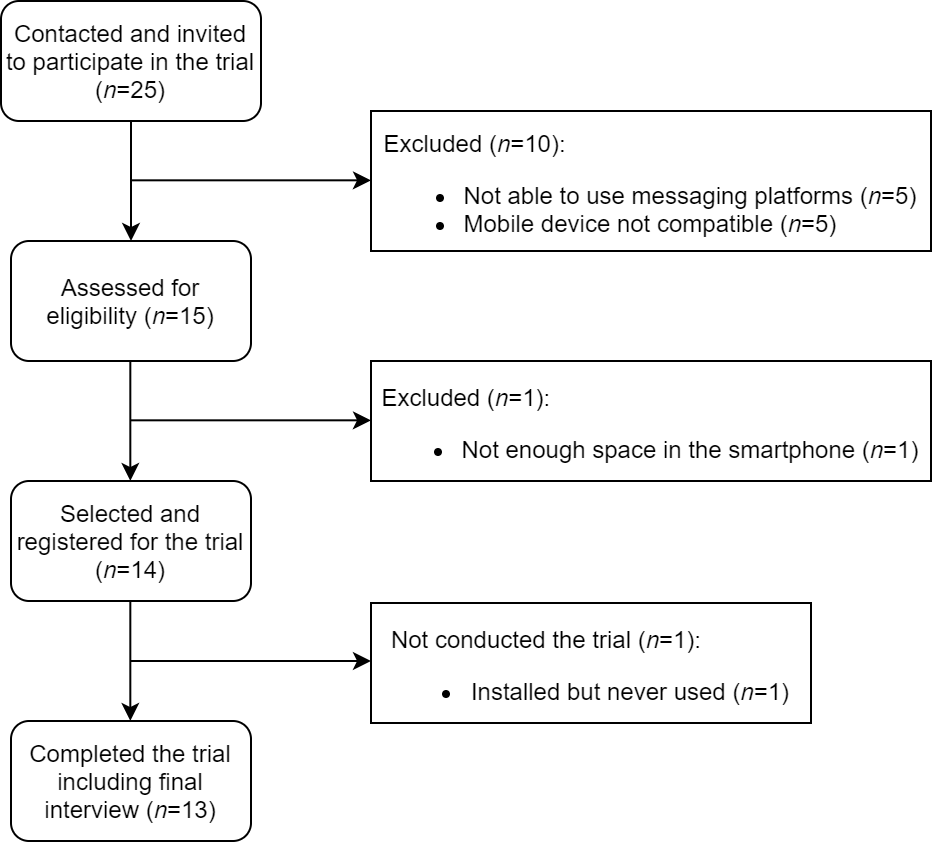
Flow diagram of patient selection and completion of the pilot study.

In total, 15 patients completed a baseline assessment for eligibility (as shown in Figure 2). One patient needed to be excluded from the pilot study because the smartphone did not have enough space to install new applications. After selecting and registering the patients for the study, one patient did not interact with the virtual assistant. Finally, a total of 5 healthcare professionals and 13 patients with type 2 diabetes mellitus and depressive disorder participated and completed the study from 9 May 2019 to 9 February 2020. The average age of patients was 63.8 years (SD 9.1) (range 44–83 years) and 69% of them were females (9/13). The patients needed to take an average of five different drugs per day. They also had periodical medical appointments every two or three months with their nurses if needed.

### 3.2. Evaluation Outcomes

#### 3.2.1. Medical Outcomes

The results related to medical outcomes are shown in Table 2 and Table 3. The average level of HbA1c improved from 7.6 (SD 0.7) to 7.3 (SD 0.8) (results significantly different). The average PHQ-9 scores improved from 13.2 (SD 6.0) to 8.6 (3.6) (results significantly different). Furthermore, the average number of medical appointments per month was reduced from 2.0 (SD 2.6) to 1.3 (SD 1.5). We obtained the MPR value dividing the number of drugs taken by the number of days for which the medication was scheduled. Every patient presents a different number of days in the MPR estimation since each patient adapted the use of the virtual assistant to his/her own medication and needs. The MPR value was capped at 100%, and the threshold for positive medication adherence was 80%. A total of 76.9% (10/13) of the patients demonstrated positive medication adherence (note that at the beginning of the study none of them showed positive medication adherence). Medication adherence of patients before and after the study was significantly different (*p* = 0.004).

#### 3.2.2. Use Outcomes

**Figure 3:**
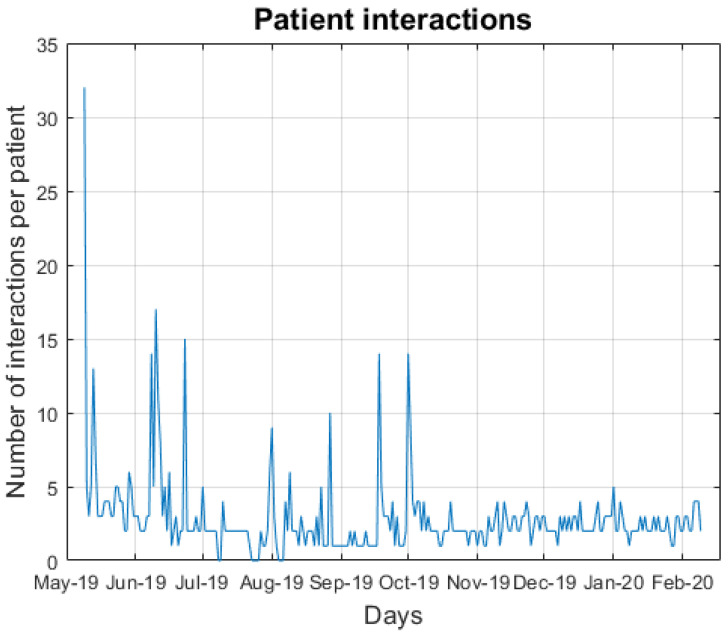
Patient interactions with the virtual assistant over time (normalized by the number of active patients).

The number of messages sent every day from the user to the virtual assistant (normalized by the number of active patients) is shown in Figure 3. The average number of messages sent per day by a patient was 2.7 (SD 2.9). A significantly high number of interactions can be observed on the first day of the study compared with the rest of the days. This is because all the participants had to take part in an initial tutorial where the basic use was explained. The rest of the peaks during the study mean that one or more patients were exploring the different options of the virtual assistant. In general, most of the patients interacted with the virtual assistant uniformly. A total of 88.5% of the interactions (4278/4835) were made using numeric-based messages, in contrast with 11.5% of text-based messages sent (557/4835).

The messages sent by the patients to the virtual assistant, classified by functionality in percentage terms, are presented in Table 4. The messages sent by the virtual assistant were not included because some reminders were not answered by the patient, meaning that the functionality was not used. It can be observed that the functionalities *Reminders* and *Medication* were the most used during the study. The patients used other functionalities, such as suggestions or medical appointments, but the prevailing usage of the virtual assistant was responding to reminders.

The average number of medication reminders configured in the virtual assistant was 3.1. The use of reminders is presented in Table 5, where the number and the percentage of the reminders answered by each patient can be observed. A total of 74.4% (2184/2936) of the reminders were answered by the patients; 23.1% of users (3/13) answered less than 50% of their reminders. Three of these patients uninstalled Signal so were unable to receive any of the reminders on their phones.

The total medication taken by the patients is also presented in Table 5. Indeed, because the medication intakes can be reported by answering the reminders and also through the medication menu, we observe that patients 10 and 13 took the medication even though they did not answer the reminders. The HbA1c, the PHQ-9 and the MPR values of these two patients improved (see Table 2 and Table 3), showing that besides the reminders, being aware of the possibility of informing the virtual assistant about the medication intakes helps in improving the adherence.

A total of 125 messages (2.6% (125/4835) of the total) sent by patients were not understood by the virtual assistant. In addition, the mean of the functionalities used by patients was 2.5 (SD 0.9) (69% of the patients (9/13) only used the functionalities *Medication* and *Reminders*, indicating that patients prefer to have as simple a virtual assistant as possible). Finally, around 23% of the patients (3/13) uninstalled Signal during the study.

### 3.3. Participant Interviews

#### 3.3.1. Intermediate Opinion Interview

The opinion interviews took place during October 2019. All patients participating in the study agreed that the virtual assistant is useful, and they also reported that it will be useful with patients older than themselves. They all used the virtual assistant daily and it covered their medication reminder needs. Around 38% of the patients (5/13) found difficulties in learning how to use the virtual assistant, such as not being comfortable with the menu system (based on options with numbers). A total of 15.4% of the patients (2/13) reported that the virtual assistant was not able to understand what they said.

All the patients agreed that the language and the vocabulary used by the virtual assistant were appropriate. However, around 15% of the patients (2/13) expressed the opinion that the clarity of the functionalities used in the virtual assistant was poor. One patient reported a problem of not knowing how to answer the reminders. The answers of the patients showed that they did not have any extra necessities apart from those provided by the virtual assistant. Additionally, one patient noted that the virtual assistant provided company in daily life and another patient said that the word size should be bigger.

Based on the answers given by the patients, we concluded that no technical changes in the virtual assistant were needed, but tutorials explaining the interaction and the interface (e.g., explaining how to make the size bigger in Signal) were needed.

#### 3.3.2. Post-Study Interview

The outcomes related to the patients’ answers are shown in Table 6 and Table 7. We can observe an overall improvement in medication management. Statistically significant outcomes related to taking the medication at the set time and taking the prescribed dosages were observed. All of the patients were already attending all of the medical appointments before the study, so no improvement was obtained. A statistically significant improvement was observed in the accommodation of medication schedules to their daily lives (from 3.6 to 4.1), whereas a slight improvement (not significantly different) was observed in the supervision of their family or friends. There was a notable improvement from 4.2 to 5 (all patients answered always) for completing the medical treatment without supervision and a decrease from 2.8 to 2.1 in the difficulty of remembering to take the medicines. Both outcomes were significantly different as it is shown in Table 6.

The outcomes related to the healthcare professionals’ answers are shown in Table 8 and Table 9. In the comments, the healthcare professionals mentioned that the virtual assistant sometimes failed, creating uncertainty and mistrust. One nurse also noted that the messages about the weather forecast were repetitive and created a lack of interest. Finally, one of the nurses mentioned that some patients found the process of saying that they had taken the medication of the day even if they had not answered the reminder to be too long.

## 4. Discussion

### 4.1. Principal Findings

The medical outcomes of the study show a moderate effect in the improvement of HbA1c, with a difference in the mean of −0.3%. This result is similar to the finding obtained by Cui et al. [23], where a difference in the HbA1c mean of −0.4% is observed. Other studies related to tele-assistance systems observed a difference in the HbA1c mean of −0.22% [37] and −0.4% [38]. Most of the patients who were more active in the use of the virtual assistant showed more improvements in the HbA1c measure. Moreover, the PHQ-9 score and the number of medical appointments per month were significantly reduced, with a mean difference in PHQ-9 of −4.6 and in the medical appointments per month of −0.7. A significant improvement was also obtained in medication adherence, with 76.9% of the patients (10/13) improving their performance after the study. Trials showed that higher medication adherence was associated with fewer emergency department visits, improved glycemic control, and lower medical costs [39]. Furthermore, the complexity of the clinical cases, such as multiple health conditions, is one of the factors that may influence medication errors, being the alert systems one of the next steps in person-centered care [40].

### 4.2. Patient Acceptance

The experience with the virtual assistant seems to have been very positive, as observed in the patients’ opinions and interactions throughout the study. Almost 70% of the patients (9/13) agreed with the idea of continuing using the virtual assistant after the study. This finding is consistent with the results reported by Nadarzynski et al. [41], where the acceptability of chatbots in healthcare was 67%. While observing the acceptance rate, the human factors and usability issues need to be considered, since the average age of the patients was 63.8 years and the oldest patient participating in the study was 83 years old. Most of the patients that stopped using the virtual assistant did so at the beginning. They found the menu system of the dialogues inconvenient and had difficulties in being understood by the virtual assistant. An easier interaction (e.g., based on buttons instead of text) could be used in order to try to improve the acceptance for these patients who face difficulties in the interaction.

After studying the answers in the intermediate opinion interview, we created a YouTube channel [42] where we uploaded tutorials solving all the patients’ doubts. We noticed that the number of views of our videos are sufficiently significant (with an average of 14 views (SD 6.5)) when compared with the number of participants, suggesting a positive acceptance of the videos.

We observed different behaviors in the patients. More than half of them were able to communicate fluently with the virtual assistant. Nevertheless, a few patients found various limitations while interacting with it. An analysis of the patients with difficulties who interacted with thirty messages or more showed that one patient had problems in correctly answering the reminders. However, after the second reminder ten minutes later, the patient was able to answer the reminder correctly the second time. We noted from Table 5 that patient 9 was not answering the reminders. This was because the virtual assistant was not able to understand the patient’s messages (we observed 30 interactions that were not understood). However, patient 13 had 34 interactions not understood, but this patient was able to indicate manually that the medication had been taken.

Some patients showed that they were keen to answer the virtual assistant, which may increase the attention and the responsibility of patients in taking their medication. Other patients who usually forgot to take their medication finally remembered to take it thanks to the alert on their mobile phones.

Three of the patients yielded low MPR values, as shown in Table 3. The main reason for this result is because two of them found difficulties while interacting with the virtual assistant, and the third one was only active for a few days during some months and, finally, uninstalled Signal.

Before the study, all of the patients were attending all the medical appointments, so no improvement related to the attendance was observed. Nevertheless, a reduction in the number of medical appointments per month was found in 30.8% of the patients (4/13), who went less frequently to the consultation when compared to the beginning of the study. This reduction in medical appointments implies a reduction in associated healthcare resources.

### 4.3. Healthcare Professional Acceptance

The healthcare professionals who participated in this study found the virtual assistant useful for the patients. As observed in Table 8, a total of 80% of them (4/5) agreed that the virtual assistant is useful and easy to use. In addition, all of them believed that the virtual assistant improves the medication adherence of the patients. The main use of the virtual assistant by the healthcare professionals was to register the patients and to check if their medication was correctly configured. Moreover, 60% of them (3/5) were interested in monitoring the medication intake of the patients, using the functionalities offered by the virtual assistant. Since their use of the virtual assistant was more sporadic and they did not need to use it every day, some of the professionals found the weather forecast messages repetitive and wanted to disable them.

### 4.4. Limitations

This study has several limitations. The virtual assistant is designed to ask the patient about the total period of the medication, but we observed that some patients did not update the information after the initial configuration for several reasons, such as forgetting to do so, being unaware of needing to do so, etc. In such cases, the virtual assistant stops sending reminders about the medication and consequently patients usually forget to use the virtual assistant. In addition, the patients need to have digital literacy or have people around them that can help if they find difficulties in configuring the messaging platform, or if they accidentally disable the sound of the reminders.

## 5. Conclusions

This research provided an overall perspective of how virtual assistants can affect patients’ medication adherence, and the improvements and limitations that arose while using the virtual assistant under study. The findings of the study suggest that the use of virtual assistants can be useful and effective for improving patient medication adherence (patients answered 74.4% of the reminders received, the HbA1c mean improved 0.3%, and the PHQ-9 mean improved 4.6). The mean of the medical appointments per month decreased by 0.7 appointments per month, which supports the potential use of virtual assistants for reducing associated healthcare resources. Furthermore, our findings suggest that virtual assistants can provide a tool for healthcare professionals to help patients improve their adherence by configuring the medication themselves, or checking if the medication is correctly configured. Because of the flexibility of the chatbot architecture, the virtual assistant can easily be used for other chronic diseases, such as psoriasis and cancer, among others, and it will be studied in future works.

## Figures and Tables

**Table 1:** Description of the functionalities used in the study.

Functionality	Tasks
Reminders	1. Sends a message at the reminder time
	2. Sends three reminders, one every 10 min
	3. Provides the medication box image if selected
Medication	1. Shows the current medication
	2. Adds new medication
	3. Deletes medication
	4. Shows the drug history
	5. Informs the virtual assistant that the patient has taken the day’s medication
	6. Shows the medication intake summary
	7. Shows the remaining medication to be taken during the day
	8. Sets the reminders for taking the medication
	9. Shows the medication information (box, information pamphlet, etc.)
Suggestions	Receives feedback from users
Medical appointments	1. Shows the medical appointments
	2. Adds new medical appointments
	3. Modifies medical appointments
	4. Deletes medical appointments
	5. Sets notifications of the appointments
Tutorials	Lists the tutorials available on YouTube

**Table 2:** Level of glycosylated hemoglobin and PHQ-9 for each patient before and after using the virtual assistant.

Patient	HbA1c Values (%) (*p* = 0.02)		PHQ-9 Score (*p* = 0.002)	
	Before	After	Before	After
1 *	7.6	7.4	11	9
2	7.4	6.9	5	4
3	7.5	7.2	18	10
4	6.9	6.2	6	4
5	7.8	7.6	9	5
6	7.8	6.9	22	12
7 *	7.9	7.9	14	8
8	9.3	9.2	5	5
9	6.9	6.9	16	7
10	7.9	6.9	20	14
11 *	7.8	8.0	21	15
12	6.7	6.8	13	10
13	7.5	7.2	12	9

* Patients who had uninstalled Signal: results calculated using the period they had Signal installed.

**Table 3:** Medical appointments per month for each patient before and after using the virtual assistant and MPR values after using the virtual assistant.

Patient	Number of Medical Appointments per Month (*p* = 0.10)		MPR %
	Before	After	
1 *	2	2	100.0
2	10	6	86.3
3	1	1	19.8
4	1	1	100.0
5	4	1	100.0
6	1	1	83.0
7 *	1	1	86.7
8	1	0	91.7
9	1	1	27.1
10	2	1	92.9
11 *	0.3	0.3	16.5
12	1	1	100.0
13	1	1	100.0

* Patients who had uninstalled Signal: results calculated using the period they had Signal installed.

**Table 4:** Analysis of the functionalities.

Functionality	Messages Sent by the Patients by Functionality, % (*n*) (*N* = 4069)
Reminders	59.2 (2408)
Medication	37.9 (1543)
Suggestions	1.5 (60)
Medical appointments	1.1 (43)
Tutorials	0.4 (15)

**Table 5:** Results of reminders activity and interaction information.

Patient	Reminders Answered “Yes” or “No”), % (*n*/*N*)	Reminders Not Answered, % (*n*/*N*)	Number of Times the Medication Was Taken (by “Yes” or Manually Introduced), % (*n*/*N*)	Number of Times the Patient Was Not Understood	Total Number of Functionalities Used	Nº of Functionalities Used Every Day, Mean (SD)
1 *	89.8	10.2	90.4	5	2	1.3 (0.4)
	(150/167)	(17/167)	(151/167)			
2	85.5	14.5	85.9	31	2	0.9 (0.6)
	(194/227)	(33/227)	(195/227)			
3	17.9	82.1	20.2	1	2	0.5 (0.7)
	(15/84)	(69/84)	(17/84)			
4	94.0	6.0	95.9	4	4	1.1 (0.5)
	(638/679)	(41/679)	(651/679)			
5	88.3	11.7	87.9	0	2	1.0 (0.4)
	(218/247)	(29/247)	(217/247)			
6	80.5	19.5	81.6	0	2	0.8 (0.5)
	(140/174)	(34/174)	(142/174)			
7 *	82.8	17.2	89.7	2	2	0.9 (0.6)
	(24/29)	(5/29)	(26/29)			
8	91.0	9.0	91.0	0	2	0.8 (0.6)
	(132/145)	(13/145)	(132/145)			
9	13.5	86.5	13.5	30	4	0.4 (0.6)
	(26/192)	(166/192)	(26/192)			
10	79.9	20.1	92.9	2	2	1.1 (0.5)
	(147/184)	(37/184)	(171/184)			
11 *	11.0	89.0	16.5	0	3	0.2 (0.4)
	(10/91)	(81/91)	(15/91)			
12	90.9	9.1	91.6	15	2	1.1 (0.3)
	(261/287)	(26/287)	(263/287)			
13	53.3	46.7	83.3	35	4	1.7 (0.7)
	(229/430)	(201/430)	(358/430)			

* Patients who had uninstalled Signal: results calculated using the period they had Signal installed.

**Table 6:** Comparison between before and after using the virtual assistant.

Statement/Question	Before, Mean (SD)	After, Mean (SD)	*p*-Value
You take the medications at the set time	4.2 (0.7)	4.8 (0.4)	0.01
You take all the indicated dosages	4.5 (0.5)	4.9 (0.3)	0.02
You attend your medical appointments	5.0 (0.0)	5.0 (0.0)	-
You accommodate your medication schedules to your daily life activities	3.6 (1.0)	4.1 (0.6)	0.02
Your family or friends are involved in your care	2.2 (1.4)	2.5 (1.3)	0.15
You complete the medical treatment without supervision of your family or friends	4.2 (0.8)	5.0 (0.0)	0.02
How often is it difficult to remember that you should take all your medicines?	2.8 (1.3)	2.1 (1.3)	0.04

**Table 7:** Patients’ responses.

Question	Yes, % (*n*) (*N* = 13)	No, % (*n*) (*N* = 13)
Do you ever forget to take medications to treat your disease?	23.1 (3)	76.9 (10)
When you are well, do you stop taking your medication?	0.0	100.0
If you ever feel bad, do you stop taking it?	15.4 (2)	84.6 (11)
Did you find it easy to use the virtual assistant?	92.3 (12)	7.7 (1)
Do you think it is useful for you?	92.3 (12)	7.7 (1)
Do you think the virtual assistant improves your medication adherence?	92.3 (12)	7.7 (1)
Do you think you will continue using the virtual assistant after the project?	69.2 (9)	30.8 (4)
From a certain moment, the virtual assistant began to give the weather forecast. Has this helped you to use the virtual assistant more frequently?	100.0	0.0

**Table 8:** Healthcare professionals’ responses.

Question	Yes, % (*n*) (*N* = 5)	No, % (*n*) (*N* = 5)
Did you find the virtual assistant easy to use?	80.0 (4)	20.0 (1)
Do you think it is useful for you?	80.0 (4)	20.0 (1)
Do you think it is useful for the patients?	100.0	0.0
Do you think the virtual assistant improves the medication adherence of the patients?	100.0	0.0
Did you use the functionalities to monitor the medication intakes of your patients?	60.0 (3)	40.0 (2)
Do you think you will continue using the virtual assistant after the project?	80.0 (4)	20.0 (1)

**Table 9:** Healthcare professionals’ responses.

Question	Mean (SD)
In the medical appointment, did you observe motivation in using the virtual assistant on the part of the patients?	4.2 (1.3)
In the medical appointment, did you observe dissatisfaction with the virtual assistant on the part of the patients?	1.8 (0.8)
In the medical appointment, did you observe an improvement in the patients’ medication intake?	4.2 (0.8)

## Abbreviations

The following abbreviations are used in this manuscript:

CEICA	Comité de Ética de la Investigación de la Comunidad Autónoma de Aragón
GDPR	general data protection regulation
HbA1c	glycosylated hemoglobin
MPR	medication possession ratio
PHQ-9	patient health questionnaire
SD	standard deviation

## Appendix A. Intermediate Interview Questions

**Evaluation guide**

Evaluation guide to assess the patients’ opinions about the usability of the virtual assistant: annotate all of the patient’s comments.

1. General impression
  - Was the use and communication with the virtual assistant easy to learn?
  - Was the virtual assistant useful?
  - How frequently did you use the virtual assistant?
  - Did the virtual assistant cover the needs of the medication reminders?
  - Were the functionalities used in the virtual assistant easy to understand?
2. Problems observed
  - Was there any problem while using the virtual assistant?
  - Did the medication reminders work correctly?
  - Were the messages/the vocabulary of the virtual assistant appropriate?
3. Suggestions/improvements/opinions
  - Did any needs arise not covered by the virtual assistant?
  - Conclusions /final opinions

## Appendix B. Post-Study Interview Questions

### Appendix B.1. Patient’s Interview

You will find a set of statements below. Please express exactly what you think in each case without worrying about whether other people would agree with you. Mark with an X the box that corresponds to your particular situation:

**STATEMENTS**	**BEFORE THE VIRTUAL ASSISTANT**					**AFTER THE VIRTUAL ASSISTANT**				
	**Always**	**Almost Always**	**Sometimes**	**Rarely**	**Never**	**Always**	**Almost Always**	**Sometimes**	**Rarely**	**Never**
1. You take the medications at the set time										
2. You take all the indicated dosages										
3. You attend your medical appointments										
4. You accommodate your medication schedules to your daily life activities										
5. Your family or friends are involved in your care										
6. You complete the medical treatment without supervision of your family or friends										
7. How often is it difficult to remember that you should take all your medicines?										

**QUESTION**	**YES**	**NO**
A—Do you ever forget to take medications to treat your disease?		
B—When you are well, do you stop taking your medication?		
C—If you ever feel bad, do you stop taking it?		
D—Did you find it easy to use the virtual assistant?		
E—Do you think it is useful for you?		
F—Do you think the virtual assistant improves your medication adherence?		
G—Do you think you will continue using the virtual assistant after the project?		
H—From a certain moment, the virtual assistant began to give the weather forecast. Has this helped you to use the virtual assistant more frequently?		

**DATA**	**BEFORE**	**AFTER**
HbA1c		
Number of medical appointments per month		

Thank you for your collaboration. We hope to improve our service with your help.

### Appendix B.2. Healthcare Professional’s Interview

You will find a set of questions below. Please express exactly what you think in each case without worrying about whether other people would agree with you. Mark with an X the box that corresponds to your particular situation:

**QUESTION**	**YES**	**NO**
A—Did you find the virtual assistant easy to use?		
B—Do you think it is useful for you?		
C—Do you think it is useful for the patients?		
D—Do you think the virtual assistant improves the medication adherence of the patients?		
E—Did you use the functionalities to monitor the medication intakes of your patients?		
F—Do you think you will continue using the virtual assistant after the project?		

**QUESTION**	**All the Patients**	**Almost All the Patients**	**Some Patients**	**Almost No Patient**	**No Patient**
1. In the medical appointment, did you observe motivation in using the virtual assistant on the part of the patients?					
2. In the medical appointment, did you observe dissatisfaction with the virtual assistant on the part of the patients?					
3. In the medical appointment, did you observe an improvement in the patients’ medication intake?					

Other observations of interest:

Thank you for your collaboration. We hope to improve our service with your help.
